# Synthesis and Anticandidal Activity of New Imidazole-Chalcones

**DOI:** 10.3390/molecules23040831

**Published:** 2018-04-04

**Authors:** Derya Osmaniye, Betul Kaya Cavusoglu, Begum Nurpelin Saglik, Serkan Levent, Ulviye Acar Cevik, Ozlem Atli, Yusuf Ozkay, Zafer Asim Kaplancikli

**Affiliations:** 1 Department of Pharmaceutical Chemistry, Faculty of Pharmacy, Anadolu Universty, 26470 Eskişehir, Turkey; dosmaniye@anadolu.edu.tr (D.O.); betulkaya@anadolu.edu.tr (B.K.Ç.); bnsaglik@anadolu.edu.tr (B.N.S.); serkanlevent@anadolu.edu.tr (S.L.); uacar@anadolu.edu.tr (U.A.Ç.); zakaplan@anadolu.edu.tr (Z.A.K.); 2 Doping and Narcotic Compounds Analysis Laboratory, Faculty of Pharmacy, Anadolu Universty, 26470 Eskişehir, Turkey; 3 Department of Pharmaceutical Toxicology, Faculty of Pharmacy, Anadolu Universty, 26470 Eskişehir, Turkey; oatli@anadolu.edu.tr

**Keywords:** imidazole, anticandidal activity, ergosterol inhibition, 14-alpha demethylase, docking study

## Abstract

In the present work, 15 new 1-(4-(1*H*-imidazol-1-yl)phenyl)-3-(4-substituedphenyl)prop-2-en-1-one derivatives (**3a**–**3o**) were synthesized to evaluate their antifungal activity. Structures of newly synthesized imidazole derivatives (**3a**–**3o**) were characterized by IR, ^1^H-NMR, ^13^C-NMR, and LCMSMS spectroscopic methods. The anticandidal activity of compounds (**3a**–**3o**) against *C. albicans* (ATCC 24433), *C. krusei* (ATCC 6258), *C. parapsilosis* (ATCC 22019), and *C. glabrata* (ATCC 90030) was elucidated according to the EUCAST definitive (EDef 7.1) method. Consistent with the activity studies, **3a**–**3d** were found to be more potent derivatives with their MIC_50_ values (0.78 µg/mL–3.125 µg/mL) against *Candida* strains. Compound **3c** indicated similar antifungal activity to ketoconazole against all *Candida* species and was evaluated as the most active derivative in the series. Effects of the most potent derivatives **3a**–**3d** on ergosterol biosynthesis were observed by LC-MS-MS method, which is based on quantification of the ergosterol level in *C. krusei*. Moreover, these compounds were subjected to a cytotoxicity test for the preliminary toxicological profiles and were found as non-cytotoxic. Furthermore, docking studies for the most active derivative **3c** were performed to evaluate its binding modes on lanosterol 14-α-demethylase. In addition to in vitro tests, docking studies also revealed that Compound **3c** is a potential ergosterol biosynthesis inhibitor.

## 1. Introduction

During the last few years, there has been an increased awareness of morbidity and mortality related to invasive and systemic fungal disease due to resistant fungi and immunocompromised infections such as AIDS. Candidiasis, aspergillosis, and cryptococcosis represent the three most common invasive fungal infections and have the highest mortality rates [1,2,3]. *Cryptococcus*, *Trichosporon*, *Geotrichum*, and *Rhodotorula* species also lead to diverse occurrence and restrict clinical therapy owing to the fungus variety. However, *Candida* species have been the primary reason of fungal infections [4,5]. *Candida* spp. are presently the third-to-fourth leading reason of bloodstream infections in the USA [6]. *Candida* is generally part of the normal flora of the mouth, vagina, skin, and intestinal tract and can exist in the oral cavity in 40–60% of the population without causing any problems [7,8]. If the environment of normal flora changes in a way that encourages fungal growth, *Candida* can increase and cause infections. Currently, various antifungal drugs have been used clinically in an attempt to reduce effects of fungal infections.

**Figure 1:**
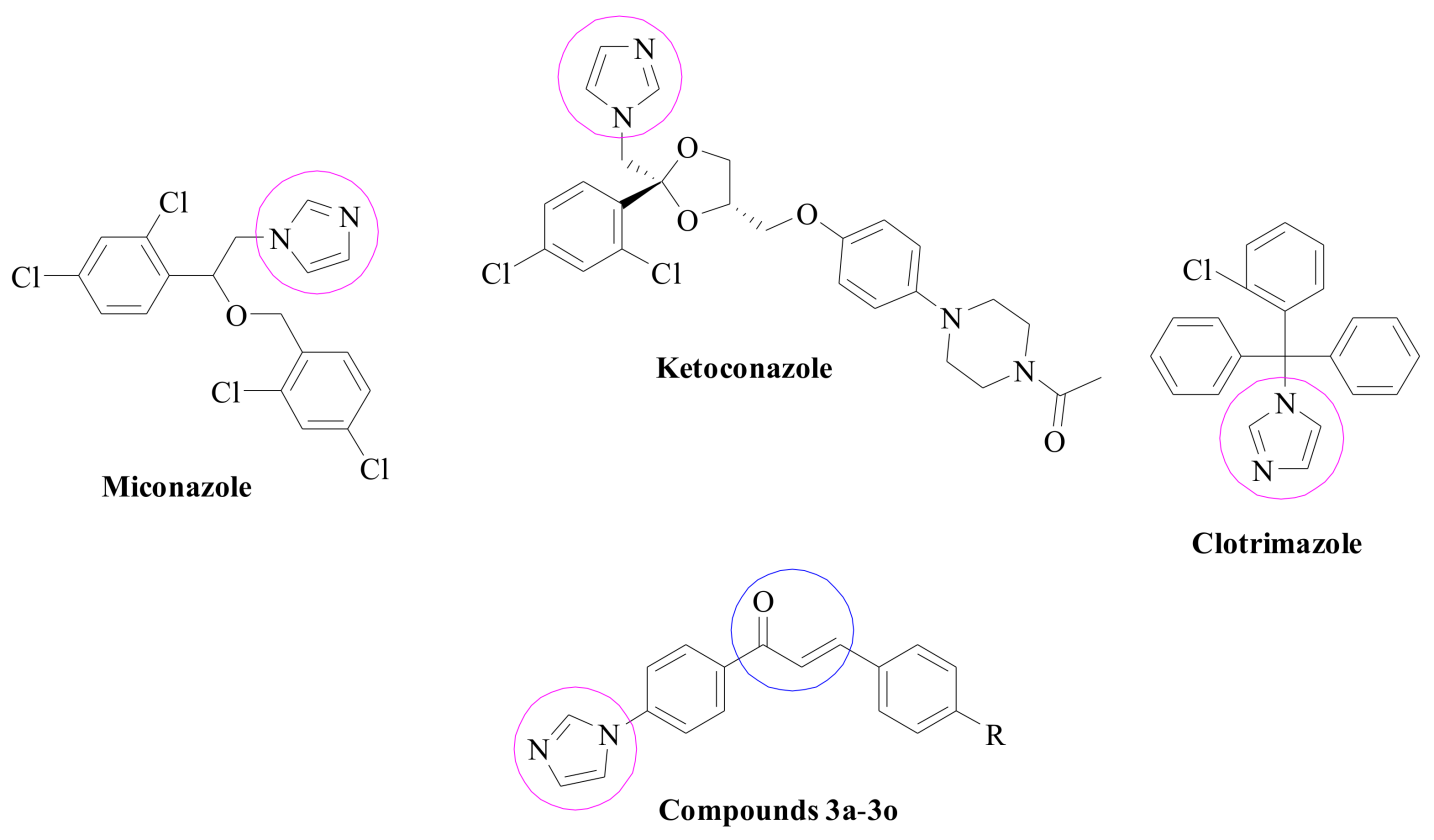
Structures of some antifungal agents and synthesized compounds (**3a**–**3o**).

The “azoles” are class of antifungal agents whose molecules are based on a pharmacophore that inhibits the activity of fungal cytochrome P45014DM (also known as lanosterol 14-α-demethylase, Erg11p, Cyp51p, and Erg16p). Azoles are administered against lanosterol 14-α-demethylase in the ergosterol pathway. Antifungal azoles bind with nitrogen atoms to iron atoms of the heme group in the target protein and block fungal membrane ergosterol biosynthesis by inhibiting the demethylation of lanosterol to ergosterol and altering the fungal membrane structure and function [9,10,11]. The variability of azole nuclei is demonstrated in their present applications as pharmaceutical drugs used in the cure of bacterial, viral, and fungal infections, worm infestations, acid reflux, cancer, inflammations, and diabetes [12]. Imidazoles, the first group to be developed in azole antifungals also block the accumulation of methylated sterols, and disrupt the ergosterol biosynthesis, which is an essential component of the fungal cell wall. Moreover, some imidazole drugs, at high concentrations, could display a direct inhibitory effect on membranes, without intervene in sterols and sterol esters [13,14,15]. Ketoconazole, miconazole, and clotrimazole are some common drugs used for the treatment of patients affected by different *Candida* species [16,17,18]. Chemical structures of these antifungal agents are summarized in Figure 1 along with compounds targeted in the present study.

In other respects, it is well known that chalcones (1,3-diaryl-2-propen-1-one) plays an important role for anticandidal activity [19,20,21,22]. Their anticandidal action has been mainly related to the reactive enone moiety. As a Michael reaction acceptor, the enone unit binds thiol groups of certain proteins. Hence, most chalcones inhibit biosynthesis of the fungal cell wall and thus clarify their antifungal potential [23].

Knowledge of antifungal activities in both functional groups (azole and chalcone) has prompted curiosity about the antifungal effects of compounds containing these two groups. Therefore, within the scope of this study, a series of new imidazole–chalcone derivatives were synthesized and evaluated for their antifungal activities.

## 2. Results and Discussion

### 2.1. Chemistry

**Scheme 1:**
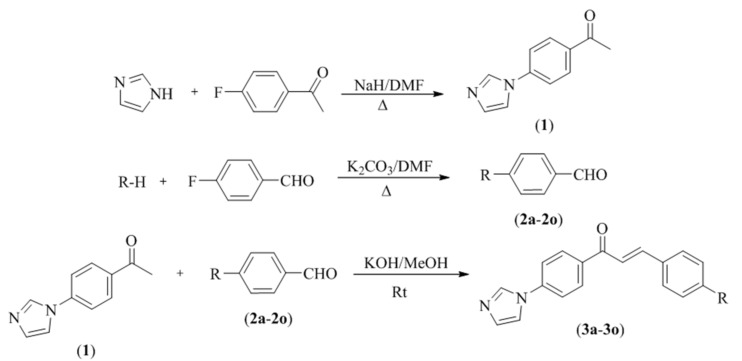
Synthesis way of the target compounds (**3a**–**3o**).

Compounds **3a**–**3o** were synthesized as summarized in Scheme 1. Initially, 4′-(imidazol-1-yl)acetophenone (**1**) was obtained under reflux by a reaction of 1-(4-fluorophenyl)ethan-1-one and 1*H*-imidazole. Secondly, 4-fluorobenzaldehyde and appropriate proton donoring group were reacted in order to obtained 4-substitutedbenzaldehydes (**2a**–**2o**). In the last step, the target compounds (**3a**–**3o**) were synthesized by means of claisen schmidt condensation using 4′-(imidazol-1-yl)acetophenone (**1**) and appropriate 4-substituted benzaldehydes (**2a**–**2o**). IR, ^1^H-NMR, ^13^C-NMR, and LCMSMS spectroscopic methods were used to perform structure elucidations of the final compounds. In the IR spectrum, C=O and C=N bonds were observed at 1601–1657 cm^−1^ and 1296–1389 cm^−1^, respectively. 1,4-Disubstituted benzene bands were observed at 808–814 cm^−1^. In the ^1^H-NMR spectrum, 1,4-disubstituted benzene protons had doublet peaks between 6.58 and 8.30 ppm. Three imidazole hydrogens gave broad singlet or triplet peaks with small coupling constant values at 7.16–7.17 ppm, 7.90–7.91 ppm, and 8.44–8.47 ppm. Vinyl protons were observed as two doublet peaks with coupling constant values, approximately 15 Hz. In the ^13^C-NMR spectrum, aromatic peaks were gained between 113 and 161 ppm. Carbonyl carbon gave a peak over 187 ppm. Aliphatic carbons belonging to substituents were observed between 12 and 64 ppm. In the mass spectrum, all masses were matched with the expected M + H values

### 2.2. Antifungal Activity

Synthesized compounds (**3a**–**3o**) were evaluated for anticandidal activity against *C. albicans* (ATCC 24433), *C. krusei* (ATCC 6258), *C. parapsilosis* (ATCC 22019), and *C. glabrata* (ATCC 90030). MIC_50_ values were determined via fluorometric measurements, using resazurin solution [24,25]. Ketoconazole and fluconazole were used as a standard drug in the activity test. Anticandidal activity results are presented in Table 1.

Consistent with the activity studies, Compounds **3a**–**3d**, were found to be more potent derivatives with their MIC_50_ values (0.78–3.125 µg/mL) against *Candida* strains. Compounds **3a**–**3c** showed higher antifungal activity against *C. krusei* with an MIC_50_ value of 0.78 µg/mL compared with standard drugs. Furthermore, MIC_50_ value of 0.78 µg/mL was recorded for Compounds **3c** and **3d** against *C. glabrata*. Compound **3c** indicated similar antifungal activity to ketoconazole and fluconazole against all *Candida* species and was evaluated as the most active derivative in the series.

Antimicrobial activity results clearly indicated that variable groups at the 4th position of the phenyl moiety have an essential impact on antifungal activity. It was observed that the presence of more liphophilic phenoxy and phenylthio groups in Compounds **3a**–**3d** have significantly enhanced anticandidal activity when compared with the compounds that carry cyclic secondary amines. It is known that lipophilicity is a key property that influences the ability of a drug to reach the target by transmembrane diffusion and to have a major effect on the biological activity [26]. Besides, augmented electron density may be another reason for better anticandidal activity due to the presence of electronically rich aromatic rings in Compounds **3a**–**3d**. Thus, it may be suggested that increased lipophilic and/or electronic characters of Compounds **3a**–**3d** caused an increase in anticandidal activity.

### 2.3. Quantification of the Ergosterol Level

Sterols are neutral lipids of eukaryotic cells, among which ergosterol is the main constituent of fungal membranes. Ergosterol has biological functions such as membrane fluidity, regulation, activity and distribution of integral membrane proteins, and control of the cell cycle [27,28]. The critical role of sterols in maintenance of cell membranes make ergosterol and its biosynthetic pathway essential for fungal growth, and a primary target for antifungal drugs to treat fungal infections [6]. From this point of view, we performed an LCMSMS (Shimadzu LCMS 8040, Kyoto, Japan) method for quantitative determination of ergosterol content of *C. krusei* as reported in our recent study [29]. Compounds **3a**–**3d**, displaying the best anticandidal activity and low cytotoxic effect, and reference agents ketoconazole and fluconazole were tested at concentrations of 0.78–3.12 µg/mL. Ergosterol standard (Product No.: 45480, Sigma-Aldrich, Darmstadt, Germany) was used for the quantification of ergostrerol in samples, which were treated with reference agents and Compounds **3a**–**3d**. Ergosterol quantity of the negative control was considered as 100%. Quadruplicate analyses were performed for all concentrations and the obtained data were expressed as mean ± standard deviation (SD) (Table 2).

According to obtained results, the decrease in ergosterol level after administration of Compounds **3a**–**3d** are noticeable when compared to the reference agents. Compounds **3a**–**3d** and reference agents significantly decreased the level of ergosterol at all tested concentrations. Therefore, it can be interpreted that Compounds **3a**–**3d** play a role in the ergosterol biosynthesis pathway.

### 2.4. Cytotoxicity Test

The main cause of failure in all stages of the new drug development process is toxicity. Early identification of toxicities of drug candidates is very important in terms of drug development studies [30]. Therefore, the MTT cell viability assay, which is suggested for cytotoxicity screening of drug candidates by ISO (10993-5, 2009) was performed [31]. Cytotoxicity of selected compounds (**3a**–**3d**), displaying strong anticandidal activity and good predicted pharmacokinetics, was determined against NIH/3T3 mouse embryonic fibroblast cell lines (ATCC CRL1658).

The cytotoxicity results of the tested compounds are presented in Table 2. IC_50_ of compounds against NIH/3T3 was much higher than their MIC_50_ values (0.78–3.125 µg/mL) against *Candida* strains. This finding shows that the antifungal activity of Compounds **3a**–**3d** is not due to general toxicity, but can be ascribed to their selective action against *Candida* species. Thus, cytotoxicity test findings enhanced the importance of Compounds **3a**–**3d** as anticandidal drug candidates.

### 2.5. Molecular Docking Studies

Docking studies were performed in order to gain more insight into the binding mode of the most active compound **3c** to lanosterol 14-α-demethylase. Lanosterol 14-α-demethylase from *Mycobacterium tuberculosis* has high homology compared with lanosterol 14-α-demethylase from *Candida* species. It has been reported that these two enzymes have a high degree of similarity between the hydrophobic cavities of the catalytic site [29,32]. Therefore, docking studies were carried out using X-ray crystal structure of lanosterol 14-α-demethylase from *Mycobacterium tuberculosis* in complex with fluconazole (PDB ID: 1EA1) [33] obtained from the Protein Data Bank server (www.pdb.org).

As stated in the antifungal activity section, Compound **3c** was found to be the most active derivative against *C. albicans*, *C. glabrata*, *C. krusei*, and *C. parapsilosis* with 1.56, 0.78, 0.78, and 0.78 µg/mL MIC values, respectively. Thus, the main purpose of docking studies was to investigate the possible interaction of this compound with lanosterol 14-α-demethylase enzyme.

**Figure 2:**
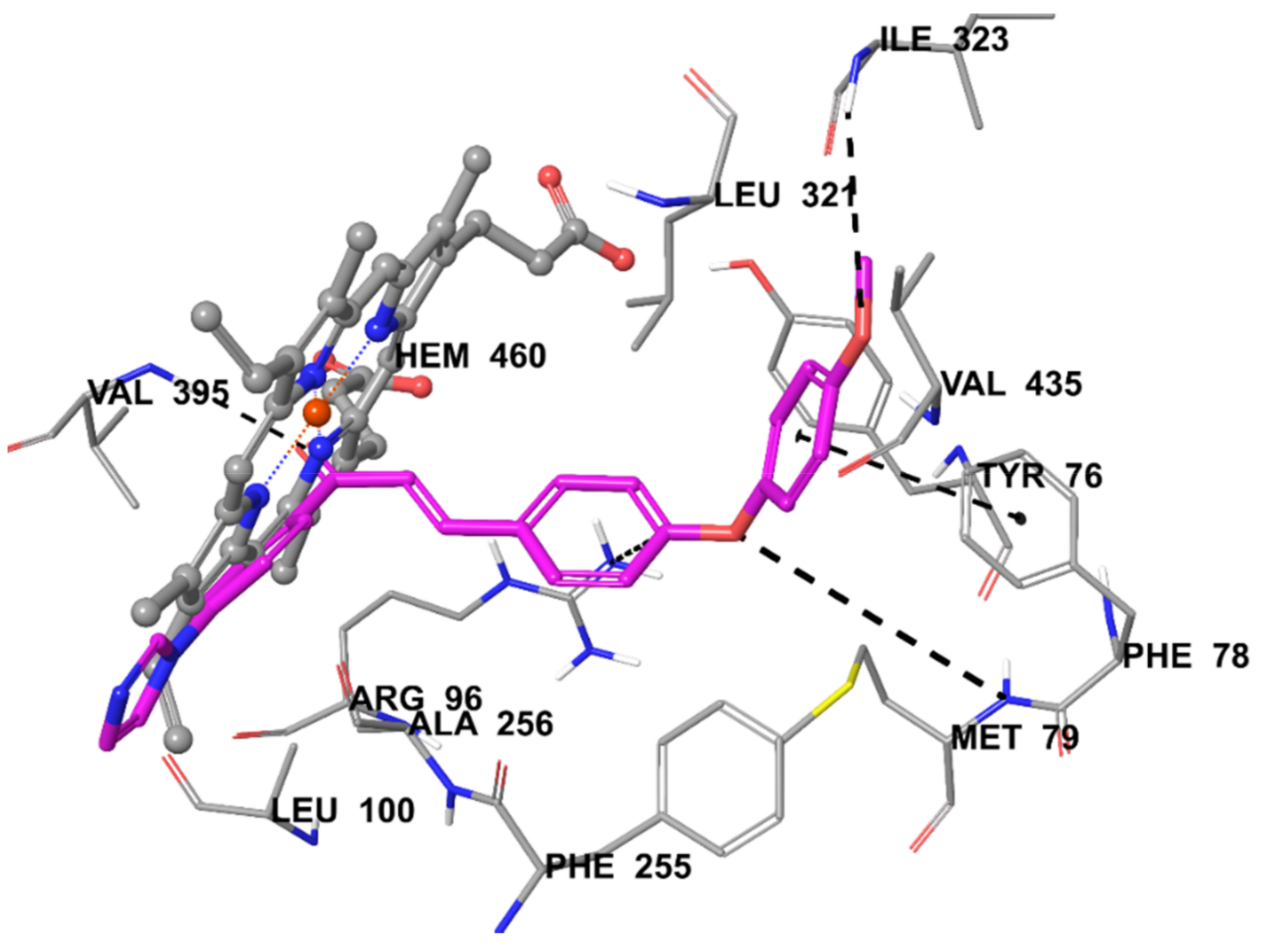
The interacting mode of Compound **3c** in the active region of 14-alpha-sterol demethylase. The inhibitor is colored with purple and HEM with grey.

The docking pose on lanesterol 14-α-sterol demethylase reveals that the interactions between Compound **3c** and HEM450 are very important in terms of binding to the active site of the enzyme (Figure 2). Imidazole and its neighboring phenyl established two π–π interactions. The other two π–π interactions were formed by other phenyl rings of the structure. Phenyl of benzylidene was in interaction with Arg96, while phenyl substituted with methoxy group created a π–π interaction with the phenyl of Phe78. Additionally, the docking pose showed that there were three hydrogen bonds providing polar interactions. Carbonyl of Compound **3c** established a hydrogen bond with the amino of Val395. Oxygen atoms of phenoxy created this interaction with the amino of Met79, whereas oxygen atoms of the methoxy group formed with the amino of Ile323. These interactions explain the stronger anticandidal activity of **3c**. It could be that C-4 of phenyl is very important in terms of binding to the enzyme active site and anticandidal activity. As a result, this additional interaction could explain the greater binding capability and stronger activity of Compound **3c** compared with other compounds.

## 3. Materials and Methods

### 3.1. Chemistry

All chemicals used in the syntheses were purchased either from Sigma-Aldrich Chemicals (Sigma-Aldrich Corp., St. Louis, MO, USA) or Merck Chemicals (Merck KGaA, Darmstadt, Germany). Melting points of the synthesized compounds were measured by MP90 digital melting point apparatus (Mettler Toledo, Columbus, OH, USA) and were presented as uncorrected. ^1^H-NMR and ^13^C-NMR spectra were recorded by a Bruker 300 MHz and 75 MHz digital FT-NMR spectrometer (Bruker Bioscience, Billerica, MA, USA) in DMSO-*d*_6_, respectively. In the NMR spectra, splitting patterns were designated as follows: s: singlet; d: doublet; t: triplet; m: multiplet. Coupling constants (*J*) were reported as Hertz. The IR spectra of the compounds were recorded using an IRAffinity-1S Fourier transform IR (FTIR) spectrometer (Shimadzu, Tokyo, Japan). LC-MS-MS studies were performed on a Schimadzu, 8040 LCMSMS spectrophotometer (Shimadzu, Tokyo, Japan). The purities of compounds were checked by TLC on silica gel 60 F254 (Merck KGaA, Darmstadt, Germany).

#### 3.1.1. Synthesis of 4′-(Imidazol-1-yl)acetophenone (**1**)

4-Fluoro acetofenone (4.976 mL, 0.041 mol), imidazole (2.789 g, 0.041 mol), and sodium hydride (NaH) (1.080 g, 0.045 mol) in DMF were refluxed for 12 h. After cooling, the mixture was poured into the ice water, and the precipitated product was washed with water, dried, and recrystallized from ethanol.

#### 3.1.2. Synthesis of Four Substituted Benzaldehydes (**2a**–**2o**)

A mixture of 4-fluoro benzaldehyde (0.259 mL, 0.002 mol), corresponding phenol, thiophenol, or amine (0.002 mol), and a catalytic quantity of potassium carbonate (K_2_CO_3_) was refluxed in DMF (20 mL) for 36 h. After completion of the reaction, the mixture was poured into ice water (50 mL), and the precipitated product was filtered, washed with deionized water, dried, and recrystallized from ethanol.

#### 3.1.3. General Procedure for the Synthesis of Target Compounds (**3a**–**3o**)

1-(4-(1*H*-imidazol-1-yl)phenyl)ethan-1-one (**1**) (0.316 g, 0.0017 mol) and appropriate 4-substituted benzaldehydes (**2a**–**2o**) derivatives (0.0017 mol) in methanol were stirred for 10 h in the presence of potassium hydroxide. The precipitated product was washed with water, dried, and recrystallized from ethanol.

*1-(4-(1H-Imidazol-1-yl)phenyl)-3-(4-(p-tolyloxy)phenyl)prop-2-en-1-one* (**3a**): Yield: 79%, M.P. = 146–148 °C, FTIR (ATR, cm^−1^): 3128 (C-H), 1653 (C=O), 1373 (C=N), 814. ^1^H-NMR (300 MHz, DMSO-*d*_6_): 2.32 (3H, s, CH_3_), 7.01 (2H, d, *J* = 8.5 Hz, disubstituted CH), 7.02 (2H, d, *J* = 8.8 Hz, disubstituted CH), 7.17 (1H, s, imidazole CH), 7.25 (2H, d, *J* = 8.2 Hz, methylphenyl CH), 7.76 (1H, d, *J* = 15.6 Hz, -HC=CH-), 7.87–7.94 (6H, m, disubstituted CH, methylphenyl CH, imidazole CH, -HC=CH-), 8.00 (1H, d, *J* = 15.6 Hz, -HC=CH-), 8.30 (2H, d, *J* = 8.8 Hz, disubstituted CH), 8.47 (1H, s, imidazole CH). ^13^C-NMR (75 MHz, DMSO-*d*_6_): δ = 20.8, 118.2, 118.3, 120.1, 120.3, 121.0, 129.8, 130.9, 131.1, 131.5, 134.0, 136.1, 136.2, 140.7, 144.1, 153.6, 160.1, 188.2. ESI-MS [M + H]^+^: 381.25 (100%).

*1-(4-(1H-Imidazol-1-yl)phenyl)-3-(4-(p-tolylthio)phenyl)prop-2-en-1-one* (**3b**): Yield: 84%, M.P. = 168–169 °C, FTIR (ATR, cm^−1^): 3120 (C-H), 1651 (C=O), 1337 (C=N), 812. ^1^H-NMR (300 MHz, DMSO-*d*_6_): 2.35 (3H, s, CH_3_), 7.17 (1H, t, *J* = 1.1 Hz, imidazole CH), 7.21 (2H, d, *J* = 8.3 Hz, disubstituted CH), 7.29 (2H, d, *J* = 7.9 Hz, methylphenyl CH), 7.40 (2H, d, *J* = 8.3 Hz, disubstituted CH), 7.72 (1H, d, *J* = 15.6 Hz, -HC=CH-), 7.84–7.88 (3H, m, methylphenyl CH, imidazole CH), 7.89–7.98 (3H, m, disubstituted CH, -HC=CH-), 8.00 (1H, d, *J* = 15.6 Hz, -HC=CH-), 8.29 (2H, d, *J* = 8.8 Hz, disubstituted CH), 8.47 (1H, s, imidazole CH). ^13^C-NMR (75 MHz, DMSO-*d*_6_): δ = 21.2, 118.3, 120.3, 122.0, 128.4, 128.9, 130.3, 130.9, 130.9, 131.0, 132.9, 133.8, 135.9, 136.2, 139.1, 140.7, 141.1, 143.9, 188.2. ESI-MS [M + H]^+^: 397.20 (100%).

*1-(4-(1H-Imidazol-1-yl)phenyl)-3-(4-(4-methoxyphenoxy)phenyl)prop-2-en-1-one* (**3c**): Yield: 92%, M.P. = 168–170 °C, FTIR (ATR, cm^−1^): 3144 (C-H), 1653 (C=O), 1342 (C=N), 814. ^1^H-NMR (300 MHz, DMSO-*d*_6_): 3.78 (3H, s, OCH_3_), 6.98 (2H, d, *J* = 8.8 Hz, methoxyphenyl CH), 7.01 (2H, d, *J* = 9.2 Hz, disubstituted CH), 7.09 (2H, d, *J* = 9.2 Hz, disubstituted CH), 7.17 (1H, t, *J* = 1.1 Hz, imidazole CH), 7.75 (1H, d, *J* = 15.5 Hz, -HC=CH-), 7.87–7.93 (6H, m, methoxyphenyl CH, disubstituted CH, imidazole CH, -HC=CH-), 8.29 (2H, d, *J* = 8.8 Hz, disubstituted CH), 8.47 (1H, t, *J* = 1.1 Hz, imidazole CH). ^13^C-NMR (75 MHz, DMSO-*d*_6_): δ = 55.9, 115.7, 117.5, 118.3, 120.3, 120.8, 121.8, 129.5, 130.9, 131.3, 131.5, 136.1, 136.2, 140.6, 144.2, 148.9, 156.6, 160.8, 188.2. ESI-MS [M + H]^+^: 397.25 (100%).

*1-(4-(1H-Imidazol-1-yl)phenyl)-3-(4-((4-methoxyphenyl)thio)phenyl)prop-2-en-1-one* (**3d**): Yield: 80%, M.P. = 177–179 °C, FTIR (ATR, cm^−1^): 3111 (C-H), 1657 (C=O), 1369 (C=N), 808. ^1^H-NMR (300 MHz, DMSO-*d*_6_): 3.81 (3H, s, OCH_3_), 7.06 (2H, d, *J* = 8.8 Hz, methoxyphenyl CH), 7.12 (2H, d, *J* = 8.4 Hz, disubstituted CH), 7.17 (1H, s, imidazole CH), 7.49 (2H, d, *J* = 8.8 Hz, methoxyphenyl CH), 7.70 (1H, d, *J* = 15.6 Hz, -HC=CH-), 7.81 (2H, d, *J* = 8.4 Hz, disubstituted CH), 7.87 (2H, d, *J* = 8.8 Hz, disubstituted CH), 7.91 (1H, t, *J* = 1.4 Hz, imidazole CH), 7.92 (1H, d, *J* = 15.6 Hz, -HC=CH-), 8.27 (2H, d, *J* = 8.8 Hz, disubstituted CH), 8.46 (1H, s, imidazole CH). ^13^C-NMR (75 MHz, DMSO-*d*_6_): δ = 55.9, 116.1, 118.3, 120.3, 121.7, 121.8, 127.2, 130.2, 130.9, 132.5, 136.0, 136.2, 136.6, 140.7, 142.6, 143.9, 160.7, 188.2. ESI-MS [M + H]^+^: 413.20 (100%).

*1-(4-(1H-Imidazol-1-yl)phenyl)-3-(4-(pyrrolidin-1-yl)phenyl)prop-2-en-1-one* (**3e**): Yield: 77%, M.P. = 186–188 °C, FTIR (ATR, cm^−1^): 3109 (C-H), 1655 (C=O), 1373 (C=N), 810. ^1^H-NMR (300 MHz, DMSO-*d*_6_): δ 1.94–1.98 (4H, m, pyrrolidine CH_2_), 3.28–3.32 (4H, m, pyrrolidine CH_2_), 6.58 (2H, d, *J* = 8.8 Hz, disubstituted CH), 7.16 (1H, s, imidazole CH), 7.63–7.73 (4H, m, disubstituted CH, -HC=CH-), 7.85 (2H, d, *J* = 8.7 Hz, disubstituted CH), 7.90 (1H, t, *J* = 1.2 Hz, imidazole CH), 8.25 (2H, d, *J* = 8.7 Hz, disubstituted CH), 8.44 (1H, s, imidazole CH). ^13^C-NMR (75 MHz, DMSO-*d*_6_): δ = 25.4, 47.8, 112.2, 115.6, 118.3, 120.2, 121.9, 130.5, 130.8, 131.6, 136.2, 136.9, 140.2, 146.2, 149.9, 187.6. ESI-MS [M + H]^+^: 344.25 (100%).

*1-(4-(1H-Imidazol-1-yl)phenyl)-3-(4-morpholinophenyl)prop-2-en-1-one* (**3f**): Yield: 79%, M.P. = 214–215 °C, FTIR (ATR, cm^−1^): 3109 (C-H), 1653 (C=O), 1337 (C=N), 812. ^1^H-NMR (300 MHz, DMSO-*d*_6_): δ 3.27 (4H, t, *J* = 4.9 Hz, morpholine CH_2_), 3.75 (4H, t, *J* = 4.9 Hz, morpholine CH_2_), 7.00 (2H, d, *J* = 8.8 Hz, disubstituted CH), 7.16 (1H, t, *J* = 1.0 Hz, imidazole CH), 7.71 (2H, d, *J* = 15.4 Hz, -HC=CH-), 7.74–7.82 (3H, m, disubstituted CH, -HC=CH-), 7.86 (2H, d, *J* = 8.8 Hz, disubstituted CH), 7.90 (1H, t, *J* = 1.2 Hz, imidazole CH), 8.25 (2H, d, *J* = 8.7 Hz, disubstituted CH), 8.44 (1H, s, imidazole CH). ^13^C-NMR (75 MHz, DMSO-*d*_6_): δ = 47.6, 63.4, 114.6, 117.9, 118.3, 120.2, 125.2, 130.7, 130.8, 131.1, 136.2, 136.6, 140.4, 145.3, 153.2, 187.9. ESI-MS [M + H]^+^: 360.25 (100%).

*1-(4-(1H-Imidazol-1-yl)phenyl)-3-(4-(piperidin-1-yl)phenyl)prop-2-en-1-one* (**3g**): Y Yield: 73%, M.P. = 198–199 °C, FTIR (ATR, cm^−1^): 3078 (C-H), 1657 (C=O), 1389 (C=N), 812. ^1^H-NMR (300 MHz, DMSO-*d*_6_): δ 1.58 (6H, s, piperidine CH_2_), 3.32 (4H, s, piperidine CH_2_), 6.95 (2H, d, *J* = 8.9 Hz, disubstituted CH), 7.16 (1H, t, *J* = 1.0 Hz, imidazole CH), 7.71–7.82 (4H, m, disubstituted CH, -HC=CH-), 7.86 (2H, d, *J* = 8.7 Hz, disubstituted CH), 7.91 (1H, t, *J* = 1.3 Hz, imidazole CH), 8.26 (2H, d, *J* = 8.7 Hz, disubstituted CH), 8.45 (1H, s, imidazole CH). ^13^C-NMR (75 MHz, DMSO-*d*_6_): δ = 24.4, 25.4, 48.5, 114.6, 117.2, 118.3, 120.2, 123.9, 130.7, 130.8, 131.3, 136.2, 136.7, 140.3, 145.5, 153.2, 187.8. ESI-MS [M + 2H]^2+^: 179.70 (100%).

*1-(4-(1H-Imidazol-1-yl)phenyl)-3-(4-(3-methylpiperidin-1-yl)phenyl)prop-2-en-1-one* (**3h**): Yield: 80%, M.P. = 163–165 °C, FTIR (ATR, cm^−1^): 3107 (C-H), 1655 (C=O), 1339 (C=N), 812. ^1^H-NMR (300 MHz, DMSO-*d*_6_): 0.92 (3H, d, *J* = 6.6 Hz, CH_3_), 1.04–1.17 (1H, m, piperidine CH), 1.43–1.80 (5H, m, piperidine CH_2_), 2.73–2.82 (1H, m, piperidine CH_2_), 3.77–3.85 (2H, m, piperidine CH_2_), 6.96 (2H, d, *J* = 9.0 Hz, disubstituted CH), 7.16 (1H, s, imidazole CH), 7.71–7.73 (4H, m, disubstituted CH, -HC=CH-), 7.86 (2H, d, *J* = 8.8 Hz, disubstituted CH), 7.91 (1H, t, *J* = 1.2 Hz, imidazole CH), 8.26 (2H, d, *J* = 8.8 Hz, disubstituted CH), 8.45 (1H, s, imidazole CH). ^13^C-NMR (75 MHz, DMSO-*d*_6_): δ = 19.6, 24.8, 30.6, 33.0, 47.9, 55.3, 114.6, 117.1, 118.3, 120.2, 123.8, 130.6, 130.8, 131.4, 136.2, 136.7, 140.3, 145.5, 152.9, 187.8. ESI-MS [M + 2H]^2+^: 186.70 (100%).

*1-(4-(1H-Imidazol-1-yl)phenyl)-3-(4-(4-methylpiperidin-1-yl)phenyl)prop-2-en-1-one* (**3i**): Yield: 77%, M.P. = 189–191 °C, FTIR (ATR, cm^−1^): 3117 (C-H), 1657 (C=O), 1339 (C=N), 812. ^1^H-NMR (300 MHz, DMSO-*d*_6_): δ 0.92 (3H, d, *J* = 6.4 Hz, CH_3_), 1.10–1.24 (2H, m, piperidine CH_2_), 1.52–1.70 (3H, m, piperidine CH_2_), 2.76–2.85 (2H, m, piperidine CH_2_), 3.87–3.91 (2H, m, piperidine CH_2_), 6.96 (2H, d, *J* = 8.9 Hz, disubstituted CH), 7.16 (1H, t, *J* = 1.0 Hz, imidazole CH), 7.71–7.74 (4H, m, disubstituted CH, -HC=CH-), 7.86 (2H, d, *J* = 8.7 Hz, disubstituted CH), 7.91 (1H, t, *J* = 1.3 Hz, imidazole CH), 8.26 (2H, d, *J* = 8.7 Hz, disubstituted CH), 8.45 (1H, s, imidazole CH). ^13^C-NMR (75 MHz, DMSO-*d*_6_): δ = 22.2, 30.8, 33.6, 47.9, 114.7, 117.2, 118.3, 120.2, 123.9, 130.7, 130.8, 131.3, 136.2, 136.7, 140.3, 145.5, 153.0, 187.8. ESI-MS [M + 2H]^2+^: 186.75 (100%).

*1-(4-(1H-Imidazol-1-yl)phenyl)-3-(4-(3,5-dimethylpiperidin-1-yl)phenyl)prop-2-en-1-one* (**3j**): Yield: 81%, M.P. = 160–161 °C, FTIR (ATR, cm^−1^): 3035 (C-H), 1622 (C=O), 1335 (C=N), 815. ^1^H-NMR (300 MHz, DMSO-*d*_6_): δ 0.90 (6H, d, *J* = 6.5 Hz, CH_3_), 1.59–1.79 (4H, m, piperidine CH_2_), 2.28–2.36 (2H, m, piperidine CH_2_), 3.85–3.90 (2H, m, piperidine CH_2_), 6.97 (2H, d, *J* = 9.0 Hz, disubstituted CH), 7.16 (1H, t, *J* = 1.0 Hz, imidazole CH), 7.70–7.73 (4H, m, disubstituted CH, -HC=CH-), 7.86 (2H, d, *J* = 8.8 Hz, disubstituted CH), 7.91 (1H, t, *J* = 1.3 Hz, imidazole CH), 8.23 (2H, d, *J* = 8.8 Hz, disubstituted CH), 8.45 (1H, s, imidazole CH). ^13^C-NMR (75 MHz, DMSO-*d*_6_): δ = 19.5, 30.5, 42.4, 54.8, 114.5, 117.1, 118.3, 120.2, 123.7, 130.6, 130.8, 131.4, 136.2, 136.7, 140.3, 145.5, 152.7, 187.9. ESI-MS [M + 2H]^2+^: 193.70 (100%).

*1-(4-(1H-Imidazol-1-yl)phenyl)-3-(4-(4-benzylpiperidin-1-yl)phenyl)prop-2-en-1-one* (**3k**): Yield: 83%, M.P. = 178–180 °C, FTIR (ATR, cm^−1^): 3062 (C-H), 1601 (C=O), 1296 (C=N), 814. ^1^H-NMR (300 MHz, DMSO-*d*_6_): δ 1.17–1.31 (2H, m, piperidine CH_2_), 1.63–1.77 (3H, m, piperidine CH_2_), 2.54 (2H, d, *J* = 7.1 Hz, CH_2_), 2.73–2.81 (2H, m, piperidine CH_2_), 3.88–3.92 (2H, m, piperidine CH_2_), 6.96 (2H, d, *J* = 9.0 Hz, disubstituted CH), 7.16–7.21 (4H, m, monosubstituted benzene CH, imidazole CH), 7.27–7.29 (2H, m, monosubstituted benzene CH), 7.71–7.74 (4H, m, disubstituted CH, -HC=CH-), 7.86 (2H, d, *J* = 8.8 Hz, disubstituted CH), 7.91 (1H, t, *J* = 1.4 Hz, imidazole CH), 8.26 (2H, d, *J* = 8.8 Hz, disubstituted CH), 8.45 (1H, t, *J* = 1.0 Hz, imidazole CH). ^13^C-NMR (75 MHz, DMSO-*d*_6_): δ = 31.5, 37.8, 42.7, 47.8, 114.7, 117.2, 118.3, 120.2, 123.9, 126.3, 128.6, 129.5, 130.7, 130.8, 131.3, 136.2, 136.7, 140.3, 140.6, 145.5, 152.9, 187.9. ESI-MS [M + 2H]^2+^: 224.75 (100%).

*1-(4-(1H-Imidazol-1-yl)phenyl)-3-(4-(4-methylpiperazin-1-yl)phenyl)prop-2-en-1-one* (**3l**): Yield: 82%, M.P. = 205–207 °C, FTIR (ATR, cm^−1^): 3109 (C-H), 1655 (C=O), 1337 (C=N), 812. ^1^H-NMR (300 MHz, DMSO-*d*_6_): δ 2.22 (3H, s, CH_3_), 2.43 (4H, t, *J* = 5.0 Hz, piperazine CH_2_), 3.30 (4H, t, *J* = 5.0 Hz, piperazine CH_2_), 6.98 (2H, d, *J* = 8.9 Hz, disubstituted CH), 7.16 (1H, t, *J* = 1.2 Hz, imidazole CH), 7.72–7.76 (4H, m, disubstituted CH, -HC=CH-), 7.86 (2H, d, *J* = 8.8 Hz, disubstituted CH), 7.91 (1H, t, *J* = 1.3 Hz, imidazole CH), 8.27 (2H, d, *J* = 8.8 Hz, disubstituted CH), 8.45 (1H, s, imidazole CH). ^13^C-NMR (75 MHz, DMSO-*d*_6_): δ = 46.2, 47.3, 54.8, 114.7, 117.7, 118.3, 120.2, 124.7, 130.7, 130.8, 131.2, 136.2, 136.6, 140.4, 145.3, 153.0, 187.9. ESI-MS [M + 2H]^2+^: 187.20 (100%).

*1-(4-(1H-Imidazol-1-yl)phenyl)-3-(4-(4-ethylpiperazin-1-yl)phenyl)prop-2-en-1-one* (**3m**): Yield: 86%, M.P. = 188–189 °C, FTIR (ATR, cm^−1^): 3125 (C-H), 1653 (C=O), 1337 (C=N), 814. ^1^H-NMR (300 MHz, DMSO-*d*_6_): δ 1.03 (3H, t, *J* = 7.2 Hz, CH_3_), 2.37 (2H, q, *J* = 7.2 Hz, CH_2_), 2.48 (4H, t, *J* = 5.0 Hz, piperazine CH_2_), 3.30 (4H, t, *J* = 5.0 Hz, piperazine CH_2_), 6.99 (2H, d, *J* = 8.9 Hz, disubstituted CH), 7.17 (1H, s, imidazole CH), 7.73–7.77 (4H, m, disubstituted CH, -HC=CH-), 7.87 (2H, d, *J* = 8.8 Hz, disubstituted CH), 7.91 (1H, t, *J* = 1.3 Hz, imidazole CH), 8.28 (2H, d, *J* = 8.8 Hz, disubstituted CH), 8.46 (1H, s, imidazole CH). ^13^C-NMR (75 MHz, DMSO-*d*_6_): δ = 12.4, 47.4, 52.1, 52.6, 114.6, 117.7, 118.3, 120.2, 124.7, 130.7, 130.8, 131.2, 136.2, 136.6, 140.4, 145.4, 153.1, 187.9. ESI-MS [M + 2H]^2+^: 194.20 (100%).

*1-(4-(1H-Imidazol-1-yl)phenyl)-3-(4-(4-(2-(dimethylamino)ethyl)piperazin-1-yl)phenyl)prop-2-en-1-one* (**3n**): Yield: 78%, M.P. = 146–149 °C, FTIR (ATR, cm^−1^): 3036 (C-H), 1655 (C=O), 1300 (C=N), 812. ^1^H-NMR (300 MHz, DMSO-*d*_6_): δ 2.14 (6H, s, CH_3_), 2.33–2.44 (4H, m, CH_2_), 2.52 (4H, t, *J* = 4.7 Hz, piperazine CH_2_), 3.28 (4H, t, *J* = 4.7 Hz, piperazine CH_2_), 6.97 (2H, d, *J* = 8.9 Hz, disubstituted CH), 7.16 (1H, s, imidazole CH), 7.72–7.76 (4H, m, disubstituted CH, -HC=CH-), 7.86 (2H, d, *J* = 8.7 Hz, disubstituted CH), 7.91 (1H, t, *J* = 1.2 Hz, imidazole CH), 8.27 (2H, d, *J* = 8.7 Hz, disubstituted CH), 8.45 (1H, s, imidazole CH). ^13^C-NMR (75 MHz, DMSO-*d*_6_): δ = 40.0, 47.4, 53.3, 56.3, 57.1, 114.6, 117.6, 118.3, 120.2, 124.7, 130.7, 130.8, 131.2, 136.2, 136.6, 140.4, 145.4, 153.0, 187.9. ESI-MS [M + H]^+^: 430.35 (100%).

*1-(4-(1H-Imidazol-1-yl)phenyl)-3-(4-(4-(3-(dimethylamino)propyl)piperazin-1-yl)phenyl)prop-2-en-1-one* (**3o**): Yield: 79%, M.P. = 138–141 °C, FTIR (ATR, cm^−1^): 3086 (C-H), 1655 (C=O), 1339 (C=N), 812. ^1^H-NMR (300 MHz, DMSO-*d*_6_): δ 1.56 (2H, p, *J* = 7.3 Hz, CH_2_), 2.10 (6H, s, CH_3_), 2.20 (2H, t, *J* = 7.3 Hz, CH_2_), 2.31 (2H, t, *J* = 7.3 Hz, CH_2_), 2.47 (4H, t, *J* = 4.8 Hz, piperazine CH_2_), 3.28 (4H, t, *J* = 4.8 Hz, piperazine CH_2_), 6.97 (2H, d, *J* = 8.9 Hz, disubstituted CH), 7.16 (1H, s, imidazole CH), 7.70-7.76 (4H, m, disubstituted CH, -HC=CH-), 7.86 (2H, d, *J* = 8.7 Hz, disubstituted CH), 7.91 (1H, t, *J* = 1.2 Hz, imidazole CH), 8.27 (2H, d, *J* = 8.7 Hz, disubstituted CH), 8.45 (1H, s, imidazole CH). ^13^C-NMR (75 MHz, DMSO-*d*_6_): δ = 24.9, 45.7, 47.3, 53.0, 56.5, 57.8, 113.7, 114.6, 118.2, 120.2, 124.6, 130.7, 130.8, 131.2, 136.2, 136.6, 140.4, 145.3, 153.0, 187.9. ESI-MS [M + H]^+^: 444.35 (100%).

### 3.2. Antifungal Activity

Microbiological studies were performed according to the EUCAST definitive (EDef 7.1) method [34] for *C. albicans* (ATCC 24433), *C. krusei* (ATCC 6258), *C. parapsilosis* (ATCC 22019), and *C. glabrata* (ATCC 90030). Fluconazole and ketoconazole were used as control drugs. Two MIC readings were carried out for each chemical agent. The yeasts were maintained in RPMI after overnight incubation at 37 °C. The inocula of test microorganisms was adjusted to match the turbidity of a MacFarland 0.5 standard tube as determined with a spectrophotometer, and the final inoculum size was 0.5–2.5 × 10^5^ cfu/mL for the antifungal assay. Testing was carried out in RPMI at pH = 7, and the two-fold serial dilutions technique was applied. The last well on the microplates containing only inoculated broth was kept as a control, and the last well with no growth of microorganism was recorded to represent the MIC_50_ expressed in μg/mL. For the antifungal assays, the compounds were dissolved in DMSO. Further dilutions of the compounds and standard drugs in the test medium were prepared at the required quantities of 800, 400, 200, 100, 50, 25, 12.5, 6.25, 3.125, 1.5625, and 0.78 μg/mL concentrations RPMI. The completed plates were incubated for 24 h. At the end of the incubation, resazurin (20 µg/mL) was added into each well to control the growth in the wells. Completed plates were incubated for 2 h. MIC_50_ values were determined using a microplate reader at 590 nm excitation and 560 nm emission. Each experiment in the antimicrobial assays was replicated twice in order to define the MIC_50_ values given in Table 1.

### 3.3. Quantification of Ergosterol Level

Extraction of total sterols from *C. krusei* was performed as recorded by Breivik and Owades [35]. Quantification of ergosterol level in this extract was carried out in accordance with our recently described method [36].

### 3.4. Cytotoxicity Test

Cytotoxicity was tested using the NIH/3T3 mouse embryonic fibroblast cell line (ATCC^®^ CRL-1658™, London, UK). NIH/3T3 cells were incubated according to the supplier’s recommendations. NIH/3T3 cells were seeded at 1 × 10^4^ cells into each well of the 96-well plates. The MTT assay was performed as previously described [37,38]. The compounds were tested between 800 and 0.78 µg/mL concentrations. Inhibition % was calculated for each concentration according to the formula below, and IC_50_ values were determined by plotting a dose–response curve of inhibition % versus compound concentrations tested [39].

% inhibition = 100 − (mean sample × 100/mean solvent).

### 3.5. Molecular Docking Studies

A structure-based in silico procedure was applied to discover the binding modes of the most active compound **3c** to 14-alpha-sterol demethylase enzyme active sites. The crystal structure of the enzyme (PDB ID: 1EA1) [33], which was crystallized with the reference drug (fluconazole) of antifungal activity assay, was retrieved from the Protein Data Bank server (www.pdb.org).

The structure of the ligand was built using the *Schrödinger Maestro* [40] interface and was then submitted to the *Protein Preparation Wizard* protocol of the *Schrödinger Suite 2016 Update 2* [41]. The ligands were prepared by *LigPrep 3.8* [42] to assign the protonation states at pH 7.4 ± 1.0 and the atom types correctly. Bond orders were assigned and hydrogen atoms were added to the structures. The grid generation was formed using *Glide 7.1* [43], and docking runs were performed with the standard precision docking mode (SP).

## 4. Conclusions

In the present study, 15 new imidazole compounds that incorporate chalcone pharmacophores as antifungal agents were designed and synthesized. Activity studies revealed the potency of Compounds **3a**–**3d** as antifungal agents. Furthermore, toxicological study enhanced the biological importance of these compounds. Results of the ergosterol level quantification assay revealed that the mechanism of action of the compounds is related to the inhibition of the biosynthesis of ergosterol, a vital sterol regulating membrane fluidity, plasma membrane biogenesis, and function. The docking studies clearly explained the molecular interacting mode of Compound **3c** in the active region of 14-alpha-sterol demethylase. Consequently, all of this information may pave the way for medicinal chemists to synthesize similar compounds that have enhanced antifungal profiles.

## Figures and Tables

**Table:** 

Compounds	R
**3a**	4-methylphenoxy
**3b**	4-methylphenylthio
**3c**	4-methoxyphenoxy
**3d**	4-methoxyphenylthio
**3e**	pyrrolidinyl
**3f**	morpholinyl
**3g**	piperidinyl
**3h**	3-methylpiperidinyl
**3i**	4-methylpiperidinyl
**3j**	3,5-dimethylpiperidinyl
**3k**	4-benzylpiperidinyl
**3l**	4-methylpiperazinyl
**3m**	4-ethylpiperazinyl
**3n**	4-(2-dimethylaminoethyl)piperazinyl
**3o**	4-(3-dimethylaminopropyl)piperazinyl

**Table 1:** MIC_50_ (µg/mL) values of Compounds **3a**–**3o**.

Comp.	*C. albicans*	*C. glabrata*	*C. krusei*	*C. parapsilosis*
**3a**	3.125	3.125	0.78	0.78
**3b**	3.125	3.125	0.78	0.78
**3c**	1.56	0.78	0.78	0.78
**3d**	1.56	0.78	1.56	3.125
**3e**	50	12.50	12.50	12.50
**3f**	50	12.50	12.50	12.50
**3g**	50	12.50	25	12.50
**3h**	50	12.50	25	12.50
**3i**	50	12.50	12.50	12.50
**3j**	50	12.50	25	25
**3k**	50	25	25	12.50
**3l**	50	12.50	25	12.50
**3m**	50	12.50	12.50	25
**3n**	50	12.50	12.50	12.50
**3o**	50	12.50	12.50	12.50
Ketoconazole	0.78	1.56	1.56	1.56
Fluconazole	0.78	1.56	1.56	0.78

**Table 2:** Cytotoxic activity and ergosterol biosynthesis inhibition potency of Compounds **3a**–**3d** against NIH/3T3 cell line and *C. krusei*, respectively.

Compound	IC_50_ (µg/mL)	Inhibition of Ergosterol Biosynthesis (%)		
		0.78 µg/mL	1.56 µg/mL	3.12 µg/mL
**3a**	>500	66.19 ± 2.23	79.45 ± 3.16	86.47 ± 4.77
**3b**	>500	68.59 ± 1.98	81.62 ± 4.07	83.49 ± 3.18
**3c**	436.04 ± 1.03	71.14 ± 4.9	78.16 ± 2.70	84.28 ± 4.65
**3d**	387.64 ± 20.20	52.47 ± 1.83	67.14 ± 2.70	74.14 ± 2.21
Ketoconazole	-	60.99 ± 2.94	73.12 ± 4.16	84.56 ± 3.01
Fluconazole	-	61.74 ± 1.70	70.12 ± 3.22	82.13 ± 4.45

## Supplementary Materials

The following are available online.
