# Supplementary material for: Synthesis and Anticandidal Activity of New Imidazole-Chalcones
Source: Molecules. 2018 Apr 4;23(4):831. doi: 10.3390/molecules23040831 (PMC6017838; doi:10.3390/molecules23040831)
Supplement: Supplementary file 1 [file molecules-23-00831-s001.pdf]

SUPPORTING INFORMATION

SYNTHESIS AND ANTICANDIDAL ACTIVITY OF NEW IMIDAZOLE-CHALCONES

Derya Osmaniye[a,b], Betül Kaya Çavuşoğlu[a], Begüm Nurpelin Sağlık[a,b], Serkan Levent[a,b], Ulviye Acar Çevik[a,b], Özlem Atlı[c], Yusuf Özkay[a,b,*], Zafer Asım Kaplancıklı[a]

[a]*Department of Pharmaceutical Chemistry, Faculty of Pharmacy, Anadolu University, 26470 Eskişehir, Turkey*

[b]*Doping and Narcotic Compounds Analysis Laboratory, Faculty of Pharmacy, Anadolu University, 26470 Eskişehir, Turkey*

[c]*Department of Pharmaceutical Toxicology, Faculty of Pharmacy, Anadolu University, 26470 Eskişehir, Turkey*

* Corresponding author.

E-mail address: yozkay@anadolu.edu.tr (Y. Özkay).

Tel: +90-222-3350580/3779 *Fax*: +90-222-3350750.

Address: Anadolu University, Faculty of Pharmacy, Department of Pharmaceutical Chemistry, 26470, Eskişehir, Turkey.

DOPNALAB

	Item	Value
	Acquired Date&Time	11.10.2017 11:58:33
	Acquired by	System Administrator
	Filename	C:\Users\dopnalab\Desktop\derya\ZDO serisi\ZDO-51.ispd
	Spectrum name	ZDO-51
	Sample name	ZDO-5
	Sample ID	
	Option	
	Comment	
	No. of Scans	10
	Resolution	4 [cm-1]
	Apodization	Happ-Genzel

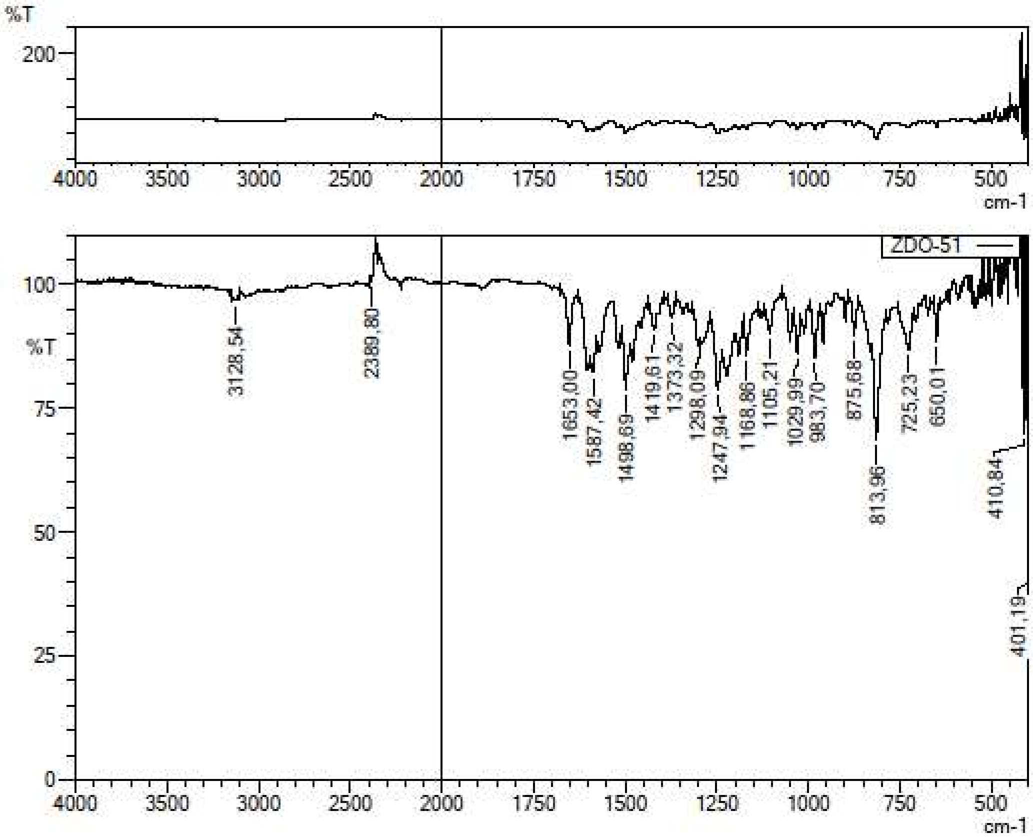

Spectra 1. IR spectra of compound **3a**

LCMSMS ANALYSES REPORT

Sample Name	:ZDO-5		
Sample ID	:		
Data Filename	: ZDO-5_002.lcd		
Method Filename	: Muratsentez.lcm		
Batch Filename	: sentez1.lcb		
Vial #	: 1-16		
Injection Volume	: 1 uL	Sample Type	: Unknown
Date Acquired	: 30.10.2017 12:56:41	Acquired by	: System Administrator
Date Processed	: 30.10.2017 12:58:43	Processed by	: System Administrator

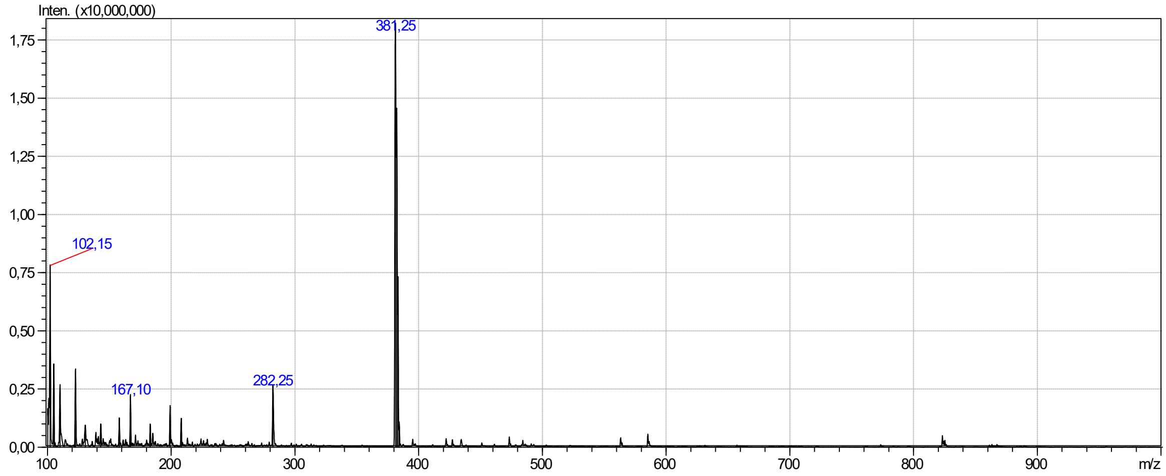

[MS Spectrum]

# of Peaks	17
Raw Spectrum	[0,034->0,507],(scan:[3->31])
Background	No Background Spectrum
Base Peak	m/z 381,25 (Inten : 18.218.219)

m/z	Absolute Intensity	Relative Intensity
102,15	7806594	42,85
167,10	2258966	12,40
282,25	2654519	14,57
381,25	18218219	100,00

Event 1

Spectra 2. LCMSMS spectra of compound **3a**

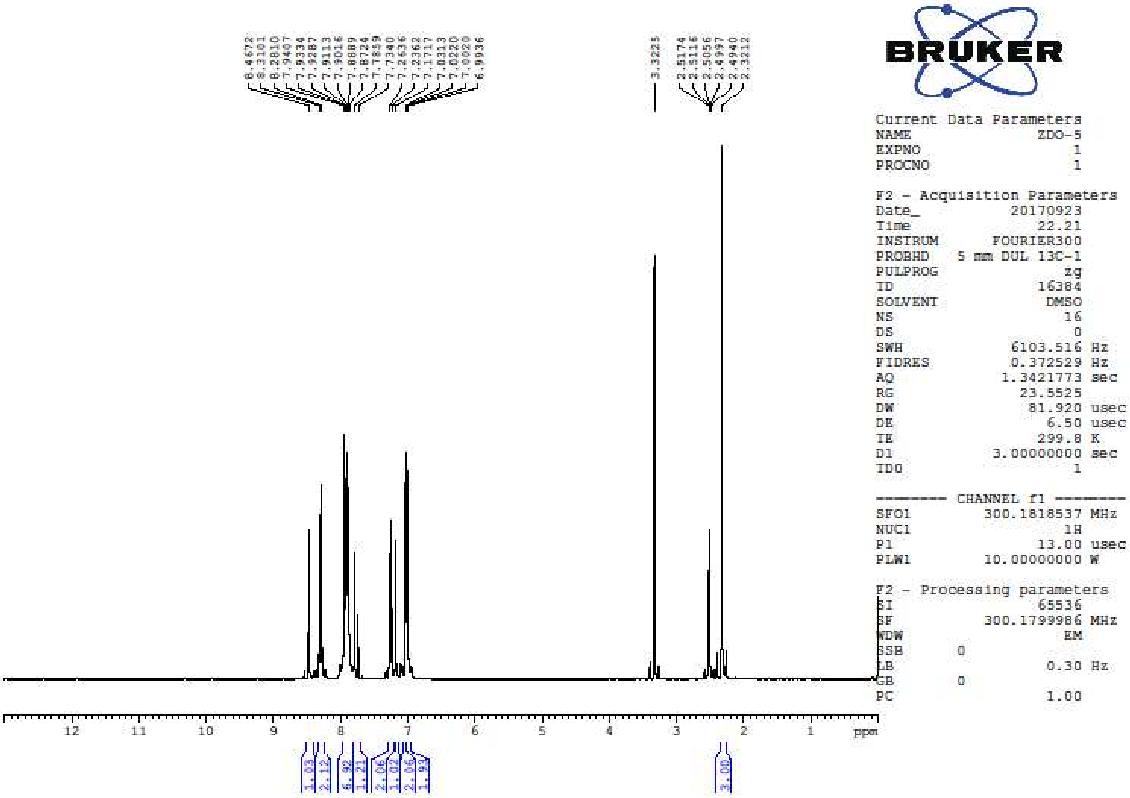

Spectra 3. ^{1}H-NMR spectra of compound **3a**

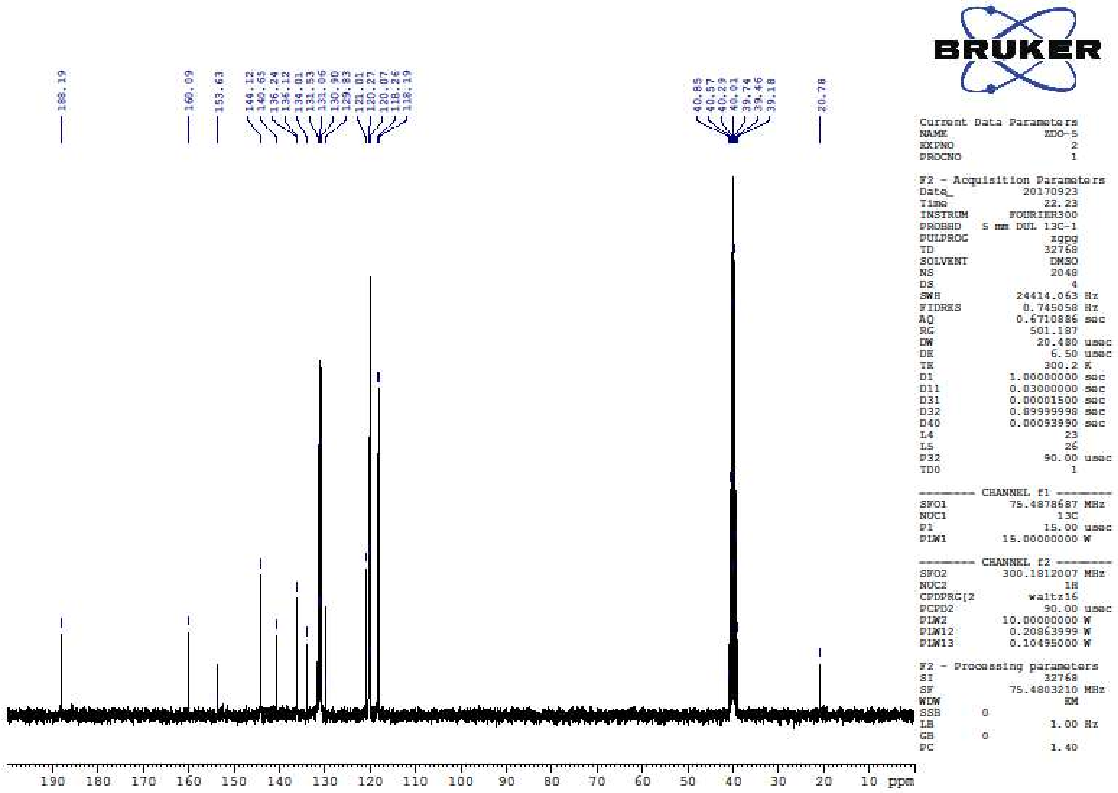

Spectra 4. ^{13}C-NMR spectra of compound **3a**

DOPNALAB

Item	Value
Acquired Date&Time	11.10.2017 12:04:45
Acquired by	System Administrator
Filename	C:\Users\dopnalab\Desktop\derya\ZDO serisi\ZDO-61.ispd
Spectrum name	ZDO-61
Sample name	ZDO-6
Sample ID	
Option	
Comment	
No. of Scans	10
Resolution	4 [cm-1]
Apodization	Happ-Genzel

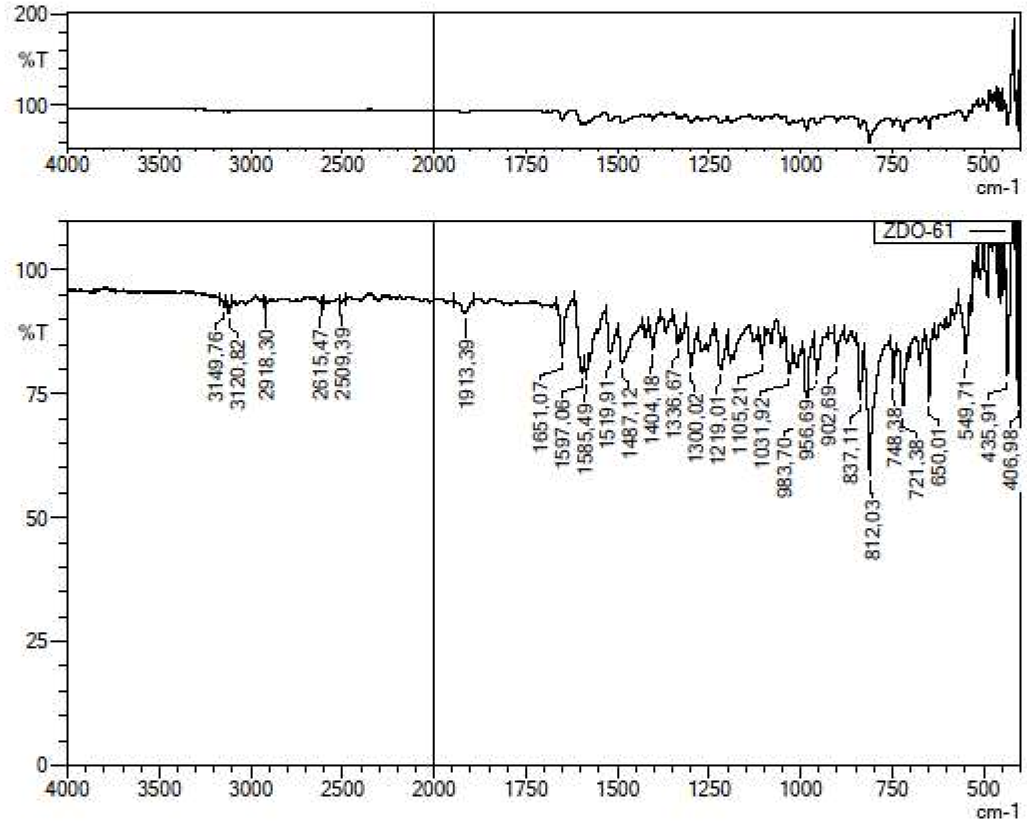

Spectra 5. IR spectra of compound **3b**

LCMSMS ANALYSES REPORT

Sample Name	:ZDO-6		
Sample ID	:		
Data Filename	: ZDO-6_003lcd		
Method Filename	: Muratsentez.lcm		
Batch Filename	: sentez1.lcb		
Vial #	: 1-17		
Injection Volume	: 1 uL	Sample Type	: Unknown
Date Acquired	: 30.10.2017 12:59:23	Acquired by	: System Administrator
Date Processed	: 30.10.2017 13:01:25	Processed by	: System Administrator

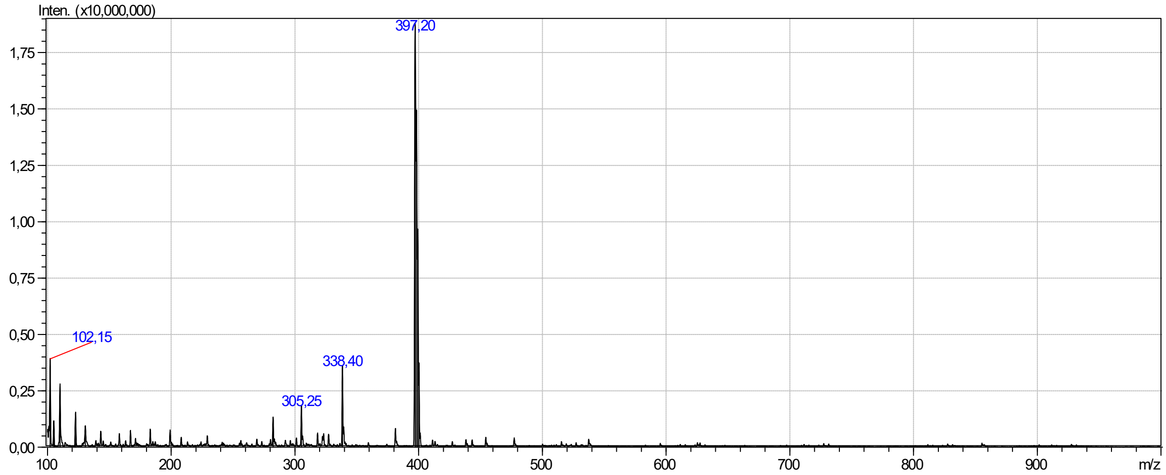

[MS Spectrum]

# of Peaks 12

Raw Spectrum [0,034->0,643],(scan:[3->39])

Background No Background Spectrum

Base Peak m/z 397,20 (Inten : 18.819.111)

m/z	Absolute Intensity	Relative Intensity
102,15	3918939	20,82
305,25	1834351	9,75
338,40	3605675	19,16
397,20	18819111	100,00
398,20	14948193	79,43

Event 1

Spectra 6. LCMSMS spectra of compound **3b**

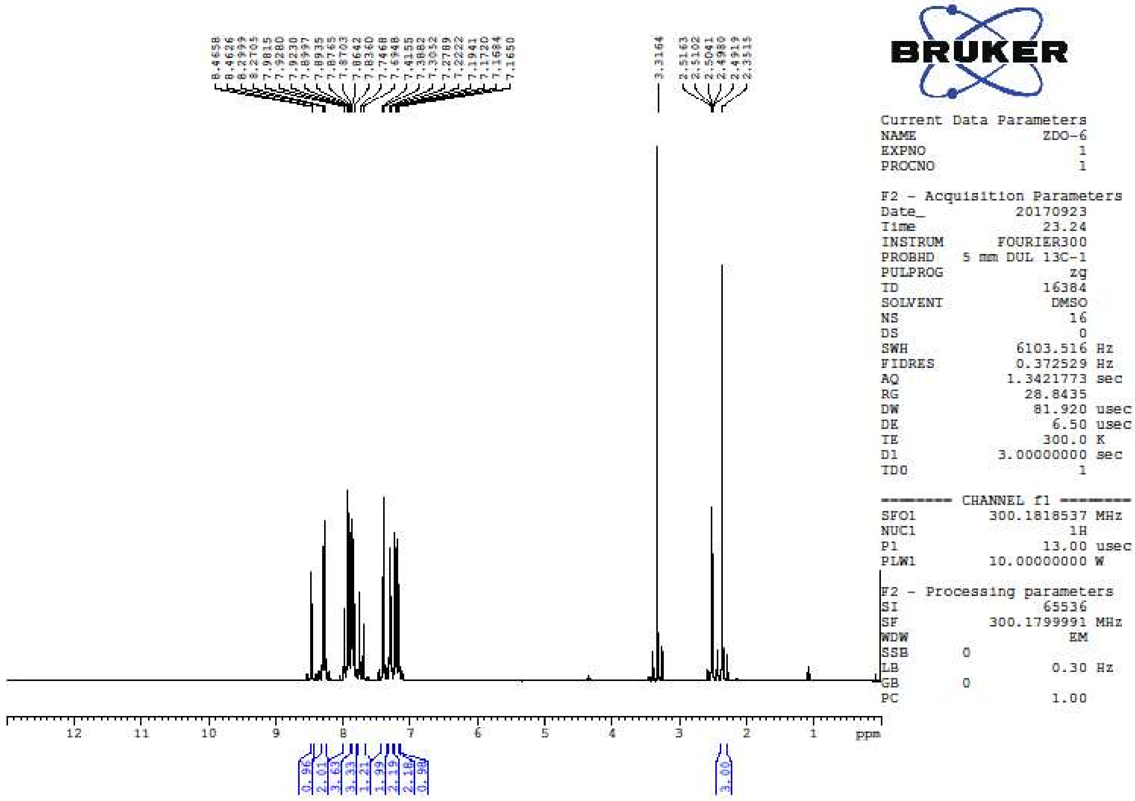

Spectra 7. ^{1}H-NMR spectra of compound **3b**

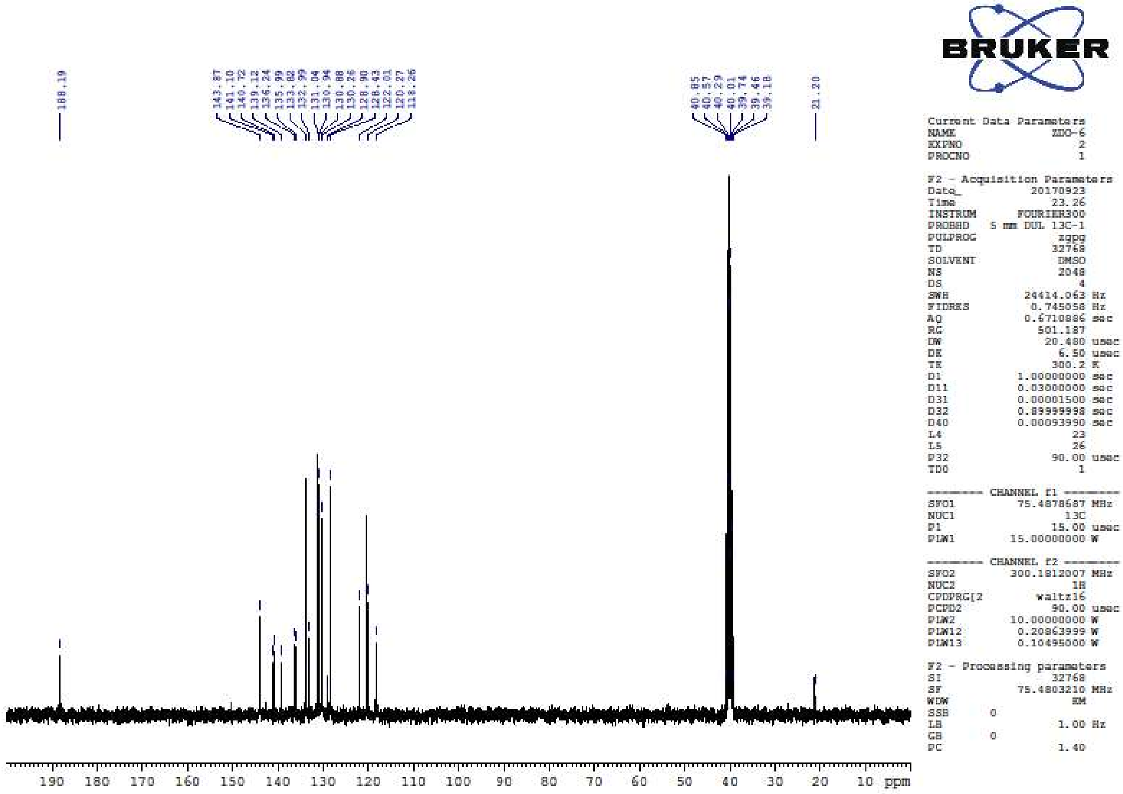

Spectra 8. ^{13}C-NMR spectra of compound **3b**

DOPNALAB

Item	Value
Acquired Date&Time	11.10.2017 12:07:05
Acquired by	System Administrator
Filename	C:\Users\dopnalab\Desktop\derya\ZDO serisi\ZDO-71.ispd
Spectrum name	ZDO-71
Sample name	ZDO-7
Sample ID	
Option	
Comment	
No. of Scans	10
Resolution	4 [cm-1]
Apodization	Happ-Genzel

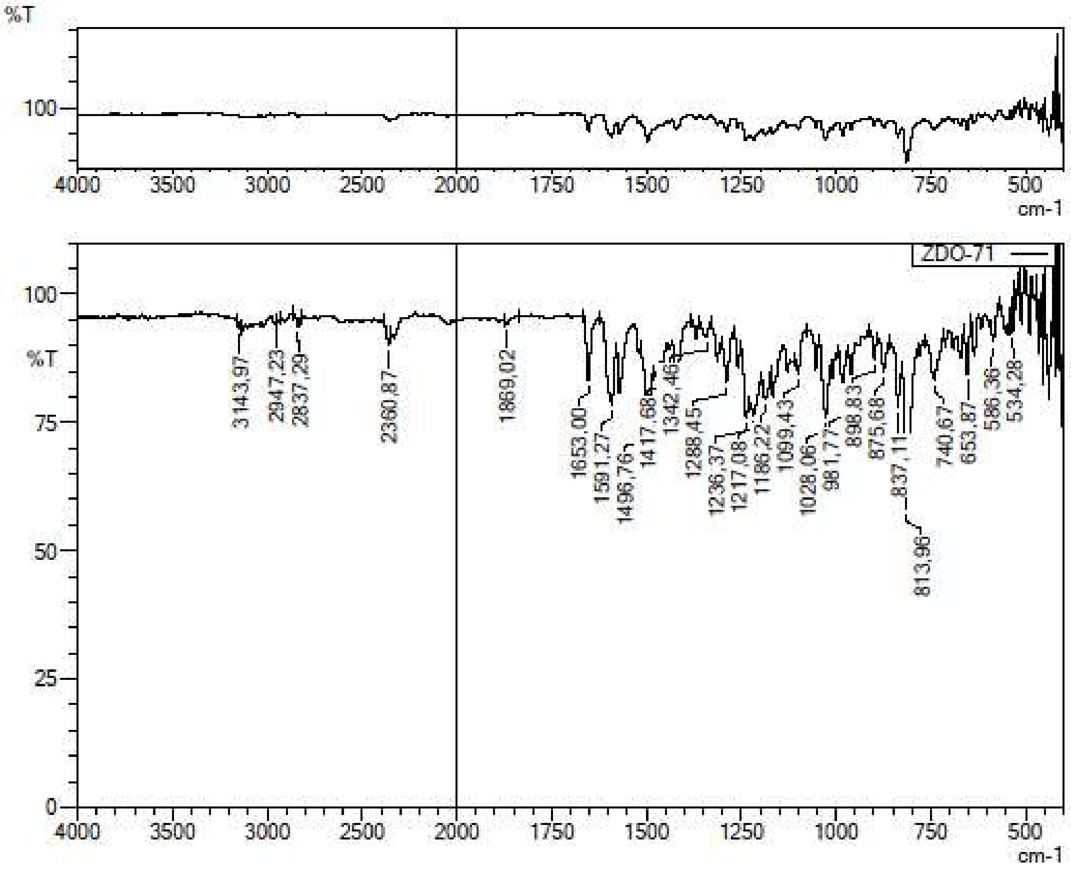

Spectra 9. IR spectra of compound **3c**

LCMSMS ANALYSES REPORT

Sample Name	:ZDO-7		
Sample ID	:		
Data Filename	: ZDO-7_004lcd		
Method Filename	: Muratsentez.lcm		
Batch Filename	: sentez1.lcb		
Vial #	: 1-18		
Injection Volume	: 1 uL	Sample Type	: Unknown
Date Acquired	: 30.10.2017 13:01:59	Acquired by	: System Administrator
Date Processed	: 30.10.2017 13:04:01	Processed by	: System Administrator

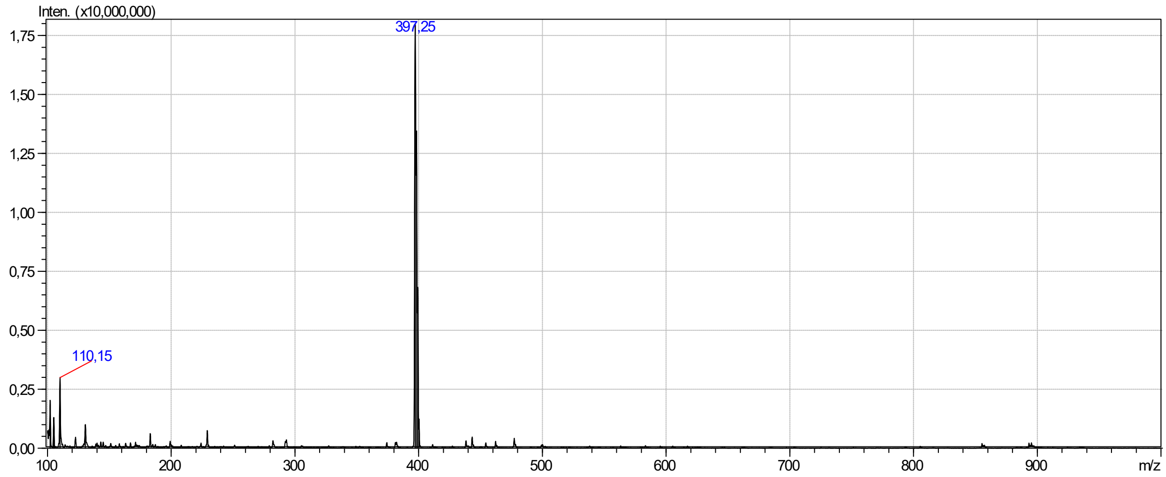

[MS Spectrum]

# of Peaks 8

Raw Spectrum [0,000->0,744],(scan:[1->45])

Background No Background Spectrum

Base Peak m/z 397,25 (Inten : 17.969.447)

m/z	Absolute Intensity	Relative Intensity
102,15	2023261	11,26
110,15	2992621	16,65
397,25	17969447	100,00
398,25	13355385	74,32
399,25	6751462	37,57
400,30	1233030	6,86

Event 1

Spectra 10. LCMSMS spectra of compound **3c**

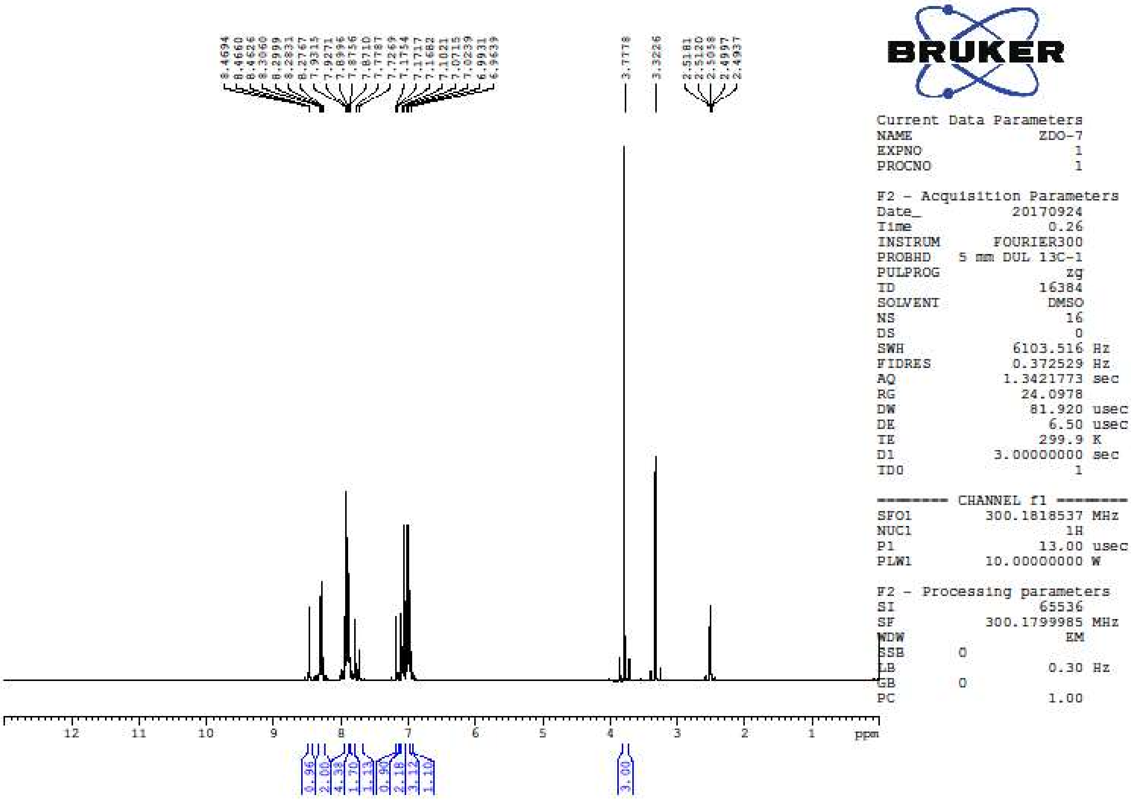

Spectra 11. ^{1}H-NMR spectra of compound **3c**

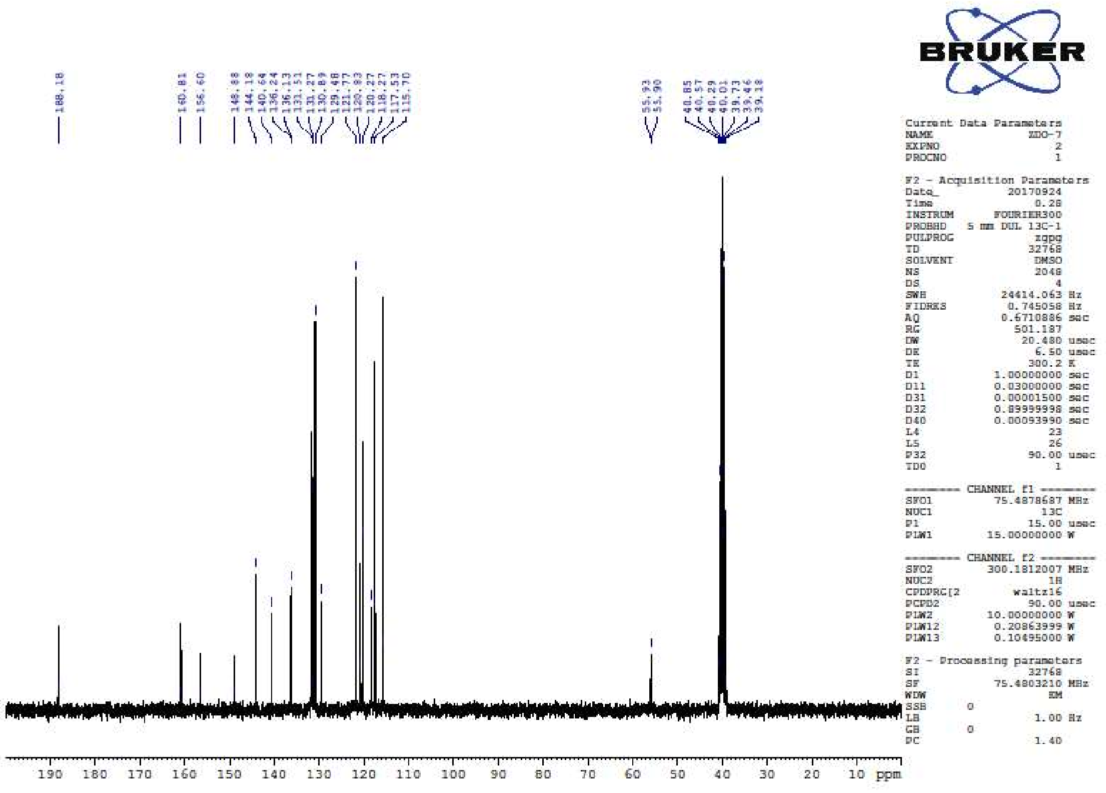

Spectra 12. ^{13}C-NMR spectra of compound **3c**

DOPNALAB

	Item	Value
	Acquired Date&Time	11.10.2017 12:09:37
	Acquired by	System Administrator
	Filename	C:\Users\dopnalab\Desktop\derya\ZDO serisi\ZDO-81.lspd
	Spectrum name	ZDO-81
	Sample name	ZDO-8
	Sample ID	
	Option	
	Comment	
	No. of Scans	10
	Resolution	4 [cm-1]
	Apodization	Happ-Genzel

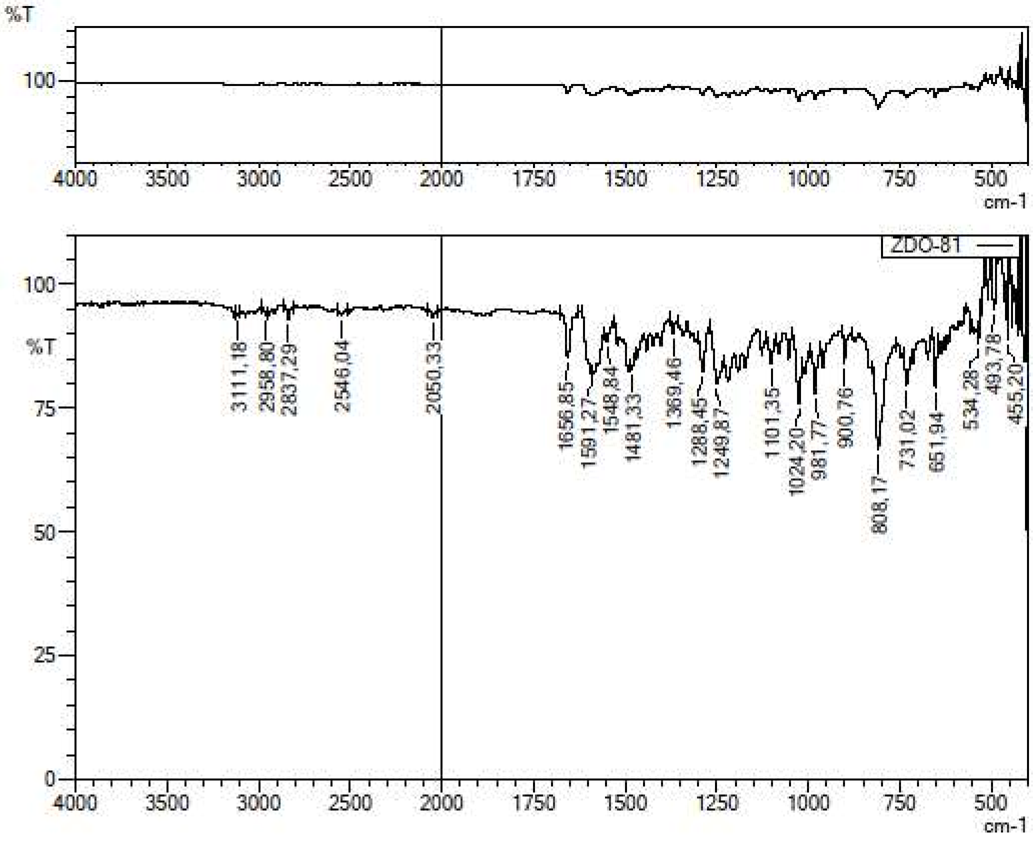

Spectra 13. IR spectra of compound **3d**

LCMSMS ANALYSES REPORT

Sample Name	:ZDO-8		
Sample ID	:		
Data Filename	: ZDO-8_005lcd		
Method Filename	: Muratsentez.lcm		
Batch Filename	: sentez1.lcb		
Vial #	: 1-19		
Injection Volume	: 1 uL	Sample Type	: Unknown
Date Acquired	: 30.10.2017 13:04:38	Acquired by	: System Administrator
Date Processed	: 30.10.2017 13:06:41	Processed by	: System Administrator

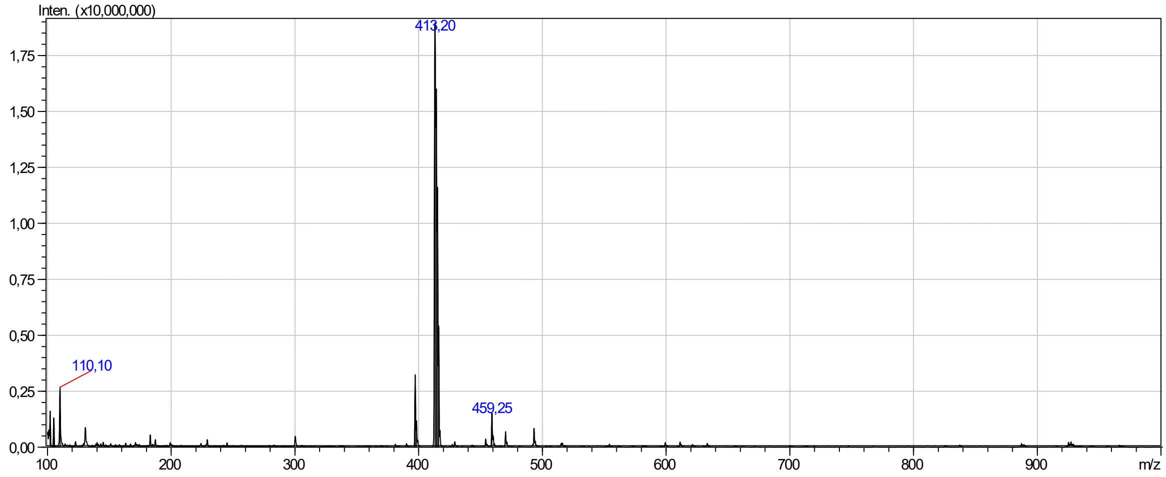

[MS Spectrum]

# of Peaks 10

Raw Spectrum [0,034->0,609],(scan:[3->37])

Background No Background Spectrum

Base Peak m/z 413,20 (Inten : 18.958.507)

m/z	Absolute Intensity	Relative Intensity
110,10	2678854	14,13
397,25	3215152	6,96
413,20	18958507	100,00
414,20	16006225	84,43
415,20	11612028	61,25
459,25	1513990	7,99

Event 1

Spectra 14. LCMSMSspectra of compound **3d**

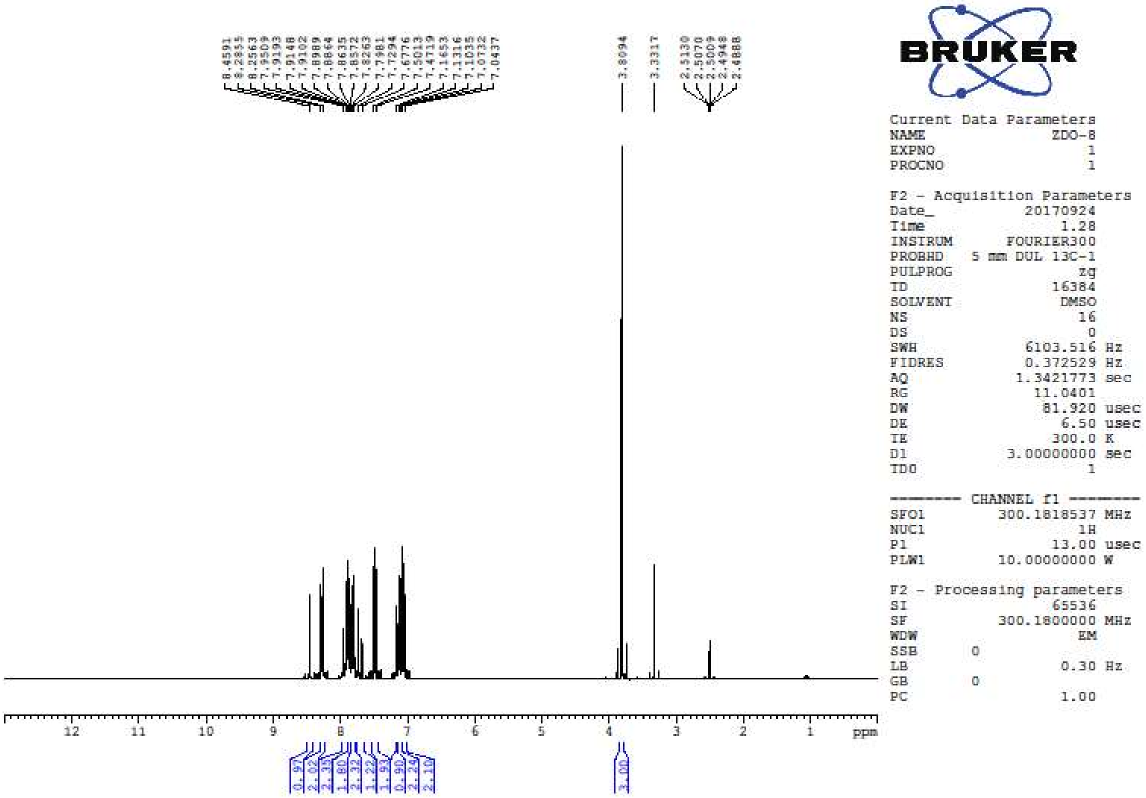

Spectra 15. ^{1}H-NMR spectra of compound **3d**

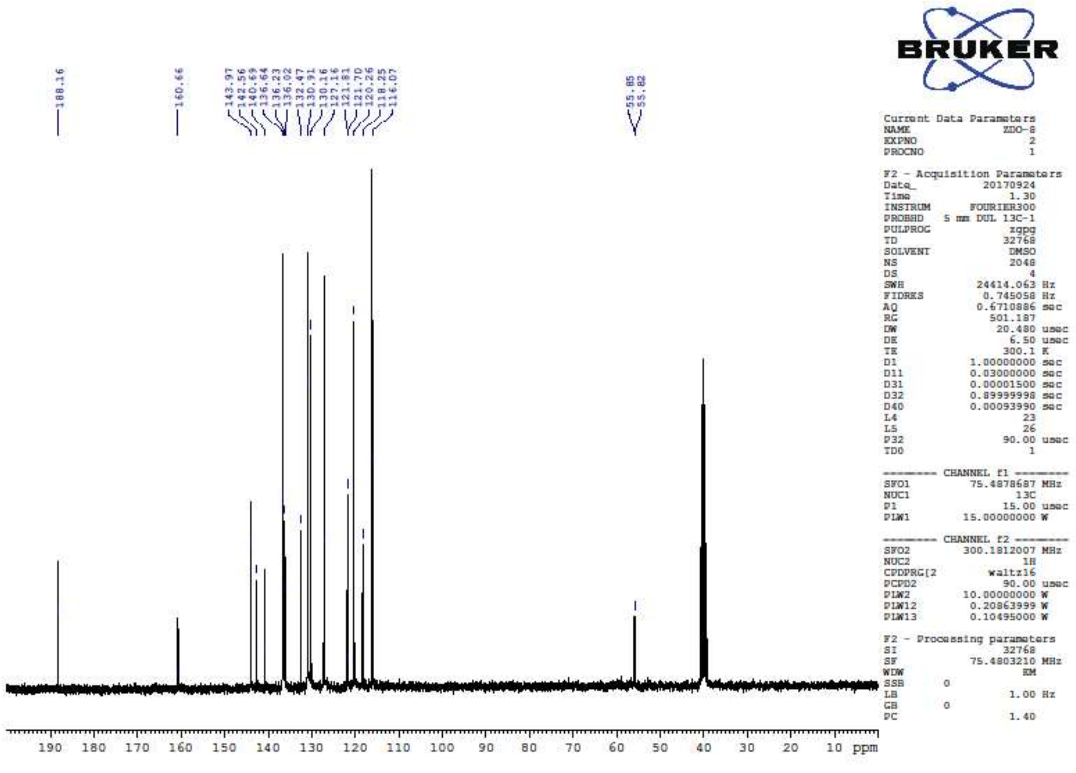

Spectra 16. ^{13}C-NMR spectra of compound **3d**
